# Comparative evaluation of YOLOv8, YOLOv11, and RT-DETR for automated microcarrier colonization assessment in bioreactor cell cultures

**DOI:** 10.3389/fbioe.2026.1849366

**Published:** 2026-07-09

**Authors:** Manuel T. Fernández de Sevilla, Raúl Valero, Beatriz Menéndez, Gonzalo García Domínguez, Cristina Blanco Sánchez, Natalia Canales, Ramón Menta Hernández, Marta H. G. Costa, Margarida Serra, Eleuterio Lombardo, Alvaro Avivar‐Valderas, Pablo Mancheño-Corvo

**Affiliations:** 1 Cell Therapies Science Department, Takeda Madrid Cell Therapy Technology Center, Madrid, Spain; 2 iBET - Instituto de Biologia Experimental e Tecnológica, Oeiras, Portugal; 3 Instituto de Tecnologia Química e Biológica António Xavier, Universidade Nova de Lisboa, Oeiras, Portugal

**Keywords:** bioreactors, cell therapy, colonization, computer vision, mesenchymal stromal cell, YOLO, in-process control

## Abstract

**Introduction:**

Monitoring microcarrier colonization during mesenchymal stromal cell (MSC) expansion in bioreactors is essential for process control. Current manual quantification methods are time-consuming, subjective, and prone to inter-operator variability. This study presents a proof-of-concept evaluation of deep learning-based object detection for automated microcarrier colonization quantification in bioreactor cell cultures

**Methods:**

Three state-of-the-art architectures—YOLOv8, YOLOv11, and RT-DETR—were compared within a unified experimental framework. A dataset of 699 fluorescence microscopy images containing 46,982 annotated microcarriers (classified as colonized or non-colonized) was used for training and evaluation. Model selection followed a three-stage Successive Halving Algorithm (SHA) strategy to efficiently identify the best-performing architecture across 12 configurations without requiring high-end computational infrastructure. COCO-pretrained transfer learning was applied across all models.

**Results:**

YOLOv8-l emerged as the top-performing model, achieving mAP50–95 of 0.855 on validation and 0.787 on test, with a colonization estimation error (Mean Absolute Error) of 11.75%. The dataset size proved sufficient for CNN-based architectures, while also revealing the higher data demands of transformer-based detectors.

**Discussion:**

These results demonstrate that automated deep learning pipelines can reliably quantify microcarrier colonization, offering a practical alternative to manual assessment in cell therapy manufacturing. The findings also suggest that newer YOLO models do not necessarily yield improvements in domain-specific biomedical tasks

## 1 Introduction

The emergence of medicinal indications of cell therapy products has increased in the last 10 years and the trend continues to continue growing until 2030 (Bahari et al., 2023). The necessity to scale production has promoted new manufacturing techniques, including the use of microcarriers in bioreactors, which enhances cell density for adherent cells and yields higher production compared with 2D planar culture platforms (Brandenberger et al., 2011; Costa et al., 2026). As bioprocesses become more complex and scale-dependent, the definition of well-characterized quality attributes and key performance indicators becomes increasingly critical to ensure consistent cell expansion and final product quality (Herbst et al., 2025; López-Fernández et al., 2024). However, this increase in the number of technical improvements has not always been accompanied by corresponding advances in cell monitoring within these systems (Stephenson and Grayson, 2018), creating a growing gap between process sophistication and the analytical tools available to monitor it in real time.

Having well defined bioreactor quality attributes and key performance indicators are fundamental to predict bioreactor and cell behavior during expansion (Herbst et al., 2025). Moreover, operating under Good Manufacturing Practice (GMP) would ensure the production of clinical-grade cells with enough quality to be granted safely to the patient (Elseberg et al., 2015). One of the critical stages of bioreactor used to expand adherent cells in microcarriers is the seeding and microcarriers colonization in early phases of cell expansion (López-Fernández et al., 2024). This step is crucial for later cell expansion and ultimately determines the final harvest yield (Derakhti et al., 2019). So, monitoring colonization in first stages of bioreactor expansion is important. Currently, colonization is measured as the percentage of microcarriers with at least one cell adhered. This calculation is normally performed by staining the cells adhered to the microcarriers with fluorescein diacetate and propidium iodide and counting manually the images taken with fluorescence-inverted microscope (Tsai and Pacak, 2021). A laboratory technician requires around 10 minutes to evaluate a single image, and at least three images per day are analyzed to obtain an average colonization percentage, translating into approximately 30 minutes of manual work per bioreactor culture day. Furthermore, inter-operator variability in manual cell counting has been reported to exceed 15% depending on operator training and fatigue (Cai et al., 2024; Salinas et al., 1997). This process is repetitive, time-consuming and entirely dependent on the operator´s expertise, introducing subjectively and potential inter-operator variability, which motivates the development of automated computer vision pipelines capable of delivering rapid, objective, and reproducible quantification of the colonization, that also aligns with regulatory expectations under Good Manufacturing Practice (GMP).

In recent years, deep learning has transformed microscopy image analysis, enabling significant progress in cell detection, colony counting, and automated quantification across biomedical domains (Choudhry, 2016; Klett et al., 2025). Object detection models have gained particular traction for their ability to simultaneously localize and classify structures of interest in a single forward pass, making them well-suited for real-time laboratory applications. Reviews of computer vision in microscopy highlight both accuracy improvements and persistent challenges related to dataset size, domain shift, and the scarcity of independent validation studies (Galata et al., 2021; Jacobs et al., 2022). Within object detection, the YOLO (You Only Look Once) family has become widely adopted in biomedical applications, from histopathology and cytology to cell culture monitoring, owing to its real-time inference speed and competitive accuracy (Heo et al., 2017). However, YOLO models are not without limitations in biomedical context. Their performance can be sensitive to domain shift when applied to imaging conditions that differ from those in the training data. Additionally, the relatively small size of most biomedical datasets increases the risk of overfitting, particularly for larger model variants, making transfer learning strategies and rigorous validation across independent datasets essential prerequisites for reliable deployment (Galata et al., 2021; Jacobs et al., 2022). These considerations motivated the systematic evaluation and threshold calibration approach adopted in the present study. Regarding the YOLO family, two versions were evaluated: YOLOv8 and YOLOv11.

YOLOv8, released by Ultralytics in early 2023, introduced anchor-free detection heads and refined training pipelines, rapidly becoming a *de facto* baseline across diverse applications despite lacking a formal peer-reviewed publication (Reis et al., 2024). Early adopters have reported successful deployment of YOLOv8 in biomedical imaging tasks including parasite detection, plant phenotyping, and cancer cell identification (Hassan et al., 2025; Sazak and Kotan, 2025; Sun et al., 2025).

Building on this trajectory, YOLOv11 was released in late 2024 with architectural refinements including C3k2 blocks and C2PSA attention modules, aimed at improving the accuracy-efficiency tradeoff (Khanam and Hussain, 2024). Preliminary benchmarks on standard datasets suggest improved mean Average Precision (mAP) at comparable or reduced parameter counts. However, independent evaluations in biomedical contexts remain scarce, leaving open the question of whether these general-domain improvements translate to specialized tasks such as microcarrier colonization assessment.

Complementing CNN-based approaches, transformer architectures have emerged as a promising alternative for object detection. The Real-Time Detection Transformer (RT-DETR), developed by Baidu, eliminates non-maximum suppression through end-to-end detection and introduces an efficient hybrid encoder for multi-scale feature processing (Zhao et al., 2024). RT-DETR achieves competitive results on COCO benchmarks with tunable accuracy-latency tradeoffs, though its performance on small biomedical datasets with domain-specific characteristics remains largely unexplored. Among available transformer-based detectors, RT-DETR was selected over other DETR variants due to its real-time inference capability—unlike original DETR architectures which suffer from slow convergence and high computational cost—and its native integration within the Ultralytics framework, which ensured consistent training pipelines, augmentation strategies, and evaluation procedures across all compared architectures, enabling a fair and reproducible comparison.

To address these gaps, the present study comparatively evaluates three state-of-the-art object detection architectures—YOLOv8, YOLOv11, and RT-DETR—for automated quantification of microcarrier colonization in MSC bioreactor cultures. These architectures were selected to represent complementary methodological paradigms: YOLOv8 as a consolidated CNN-based baseline widely adopted in biomedical imaging, YOLOv11 as its most recent official evolution allowing assessment of whether general-domain architectural improvements transfer to specialized tasks, and RT-DETR as a transformer-based alternative enabling evaluation of attention-based detection in a low-data biomedical regime. A detailed justification of model selection criteria is provided in Section 2.3.6.

Using a dataset of 699 fluorescence microscopy images containing 46,982 annotated microcarriers, we evaluate detection accuracy (mAP), colonization estimation error (MAE), and computational efficiency across 12 model configurations. A Successive Halving Algorithm (SHA) is employed for principled model selection under computational constraints.

The main contributions of this work are:

- The first comparative evaluation of YOLOv8, YOLOv11, and RT-DETR for microcarrier detection in bioreactor cell cultures.
- Evidence that newer model versions (YOLOv11) do not necessarily outperform their predecessors (YOLOv8) in domain-specific biomedical applications.
- Demonstration that transformer-based detectors (RT-DETR) underperform CNN-based approaches on small-scale microscopy datasets.
- A validated pipeline achieving <12% mean absolute error in colonization percentage estimation, suitable for routine laboratory use.

## 2 Materials and methods

### 2.1 Dataset

The dataset used in this study consisted of a total of 699 fluorescence microscopy images, acquired under controlled conditions using the green channel to visualize cell colonization within microcarriers. The images were provided by IBET (Instituto de Biologia Experimental e Tecnológica, Oeiras, Portugal) as part of cell culture research projects (Costa et al., 2026). Samples were collected from a stirred-tank bioreactor culture using adipose stromal cells (ASC) as the production cell type. Cultures were expanded in RoosterBio growth medium (RoosterBio, USA) and seeded on Synthemax II microcarriers (Corning, USA) to support adherent cell proliferation. Prior to imaging, cells were stained with fluorescein diacetate (Merck, Darmstadt, Germany) at a concentration of 20 μg/mL. Images were acquired using a fluorescence microscope (DMI6000, Leica Microsystems GmbH).

The model development and validation set was randomly split into training (70%), validation (15%), and test (15%) subsets using a partitioning script, ensuring that each image was assigned to only one subset. It is worth noting that per sampling point, three images were acquired from different fields of view within the same bioreactor, capturing distinct spatial regions of the culture. Combined with the fact that the dataset encompasses images from multiple independent bioreactors runs, different bioreactors within each run, various culture days, and different cell donors, this inherent variability minimizes the risk of data leakage. Although the random split was performed at the image level, the diversity of experimental conditions ensures that images sharing similar biological context are unlikely to dominate any single subset, and the generalization capacity of the model is therefore assessed under realistic conditions of experimental diversity. The generalization capacity of the model is therefore assessed under realistic conditions of experimental diversity.

Two categories were defined for analysis: FULL (colonized microcarriers, with green signal inside) and EMPTY (non-colonized microcarriers, lacking internal green signal). The exact proportion of both classes will be reported in the descriptive results section, accompanied by an illustrative figure, to document the class balance in the dataset.

Image sizes varied depending on acquisition, but all were standardized to 640 × 640 pixels via direct rescaling prior to training the YOLO models, ensuring consistency across training and evaluation. Importantly, the dataset included images from different culture batches and microscopy sessions, introducing variability in experimental conditions and reinforcing the robustness of the evaluation. All images analyzed in this study correspond exclusively to microcarriers obtained within the framework of experimental cell culture projects. That means no patient data or human/animal biological material containing sensitive information was used. Therefore, this study does not raise ethical issues related to privacy or data confidentiality.

### 2.2 Annotations procedure

**FIGURE 1:**
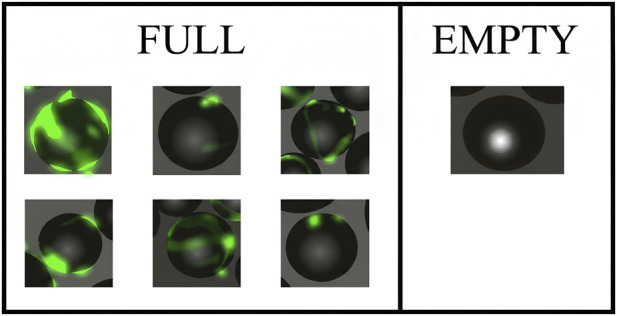
Classification of Microcarriers as Fully Colonized or Empty Based on Fluorescent Staining. Representative microscopic images used to distinguish microcarriers classified as FULL, showing clear fluorescent cell coverage (green), versus EMPTY, displaying no detectable cell attachment. These categories were used to quantify microcarrier colonization during the expansion phase.

All images were annotated by a team of five laboratory experts, who received specific training based on annotation guidelines developed for this project. These guidelines defined clear criteria for classifying microcarriers as FULL or EMPTY based on the presence or absence of internal green fluorescent signal (Figure 1). Annotation quality was further verified through an exploratory data analysis (EDA) step, which confirmed the absence of duplicate images, invalid class labels, and annotation inconsistencies across the dataset. Consistency was ensured through a consensus-based review process in which ambiguous cases were discussed and resolved collectively among the annotators. Furthermore, the binary nature of the classification task—presence or absence of fluorescent signal—inherently limits the degree of subjective interpretation, reducing the risk of systematic annotation bias.

Annotation was carried out using the open-source software LabelImg (version 1.8.6), which allowed bounding boxes to be drawn around each microcarrier and assigned the corresponding class label. As an exclusion criterion, microcarriers that appeared partially cropped at the image borders were not annotated, to avoid bias in object counting and in the estimation of colonization percentage.

Bounding box annotations were exported in YOLO format and used directly to train all detection models (YOLOv8, YOLOv11, and RT-DETR).

### 2.3 Models

#### 2.3.1 Model architectures

YOLOv8, introduced by Ultralytics in 2023, represented the first significant architectural departure from the YOLOv5 lineage (Huang et al., 2023). Its backbone relies on the C2f module, a more efficient successor of the C3 block, which improves gradient propagation and feature reuse through a split–transform–merge design with residual connections. This modification allows deeper networks without a proportional increase in computational complexity (Reis et al., 2024). The neck of YOLOv8 incorporates a Path Aggregation Network combined with Feature Pyramid Network layers, ensuring robust multi-scale feature fusion and enhancing sensitivity to small objects, which is particularly relevant in the microcarrier context where objects are compact and densely distributed (YOLOv8 Architecture, 2025). Another major innovation is the transition to an anchor-free, decoupled detection head. Unlike anchor-based heads from earlier YOLO versions, the new design predicts object centers and scales directly, reducing the need for anchor hyperparameter tuning and simplifying inference. Decoupling classification from bounding box regression also alleviates the optimization conflicts common in previous coupled architectures, improving training stability (Adeyemi, 2024). YOLOv8 optimizes bounding boxes using CIoU or DIoU Loss and enhances localization precision with Distribution Focal Loss, while classification is trained with either Binary Cross-Entropy or Focal Loss (Zheng et al., 2016). Thanks to this combination of architectural and optimization choices, YOLOv8 rapidly became one of the most widely adopted baselines across applied domains, including biomedical imaging, due to its balance of accuracy, efficiency, and deployment readiness (Terven and Cordova-Esparza, 2024).

YOLOv11, released by Ultralytics in 2024, represents the most recent official evolution of the YOLO family (Ultralytics YOLO11, 2025). While no peer-reviewed manuscript is currently available, vendor documentation reports several refinements aimed at improving accuracy and efficiency over YOLOv8. The backbone introduces enhanced convolutional modules, often described in community analyses as C3k2 blocks, designed to improve the receptive field and reduce redundancy (Ultralytics YOLO11, 2025). The neck retains the PANet/FPN fusion strategy but introduces optimized channel allocation to achieve a better trade-off between accuracy and computational cost. YOLOv11 preserves the anchor-free and decoupled head design but demonstrates improved training stability across model scales. According to the official Ultralytics benchmarks, YOLOv11 achieves higher mean Average Precision (mAP) than YOLOv8 at comparable input resolutions while using fewer parameters and FLOPs. For instance, certain variants achieve similar or superior accuracy to their YOLOv8 counterparts with reductions of up to 40% in parameter count (Jegham et al., 2025). These characteristics make YOLOv11 the state-of-the-art iteration of the YOLO family, and its inclusion in our study allows for the assessment of whether these reported improvements are maintained in biomedical microscopy tasks (Mao and Hong, 2025).

RT-DETR, proposed by Baidu in 2023, departs from convolution-only designs and implements a transformer-based detection pipeline (Wang et al., 2024). Unlike classical detectors, RT-DETR employs a hybrid encoder composed of lightweight convolutional layers followed by transformer blocks, which are then processed by a transformer decoder to produce object queries. A key innovation is the IoU-aware query selection strategy, which pre-selects high-quality object queries before decoding, enhancing both efficiency and detection precision (Wang et al., 2024). Additionally, dense positive auxiliary losses accelerate convergence and stabilize training. As with other DETR-like architectures, RT-DETR is fully end-to-end and removes the need for non-maximum suppression, a heuristic still required by YOLO models (Wang et al., 2024). This architecture has been shown to achieve accuracy comparable to DETR variants while significantly reducing inference latency, thereby providing a compelling non-CNN alternative for real-time detection tasks. By including RT-DETR in this comparison, we aimed to evaluate whether a transformer-based paradigm could offer advantages in microcarrier detection relative to the YOLO family.

#### 2.3.2 Detection heads

The detection head follows the anchor-free and decoupled design introduced in YOLOv8 (Ge et al., 2025). In this formulation, bounding box regression and classification are carried out by separate branches, which improves gradient propagation and reduces conflicts between localization and class prediction. The anchor-free approach eliminates the need for predefined anchor sizes and aspect ratios, thereby simplifying training and enhancing generalization across domains (Tian et al., 2022). At each scale of the feature pyramid, the head predicts object centers and bounding box dimensions directly, together with class probabilities. This paradigm is particularly advantageous in biomedical microscopy, where object shapes are less variable and anchor tuning would otherwise be difficult to optimize. Both YOLOv8 and YOLOv11 share this detection head design, differing primarily in backbone efficiency and feature extraction, in contrast, employs a transformer-based decoder that directly outputs object queries without requiring non-maximum suppression.

#### 2.3.3 Model size and computational complexity

An important aspect when comparing object detection architectures is the balance between predictive accuracy and computational efficiency. Modern YOLO architectures are designed as scalable families, where each variant (n, s, m, l, x) represents a different trade-off between model complexity, resource requirements, and achievable performance (Terven and Cordova-Esparza, 2024). The number of trainable parameters and the number of floating-point operations per image (FLOPs) are widely reported as proxies for this trade-off, and they provide valuable information for selecting models suitable for real-world deployment in laboratory or edge computing environments.

Beyond these theoretical metrics, computational efficiency plays a critical role in the viability of the model development process itself. Training and optimizing deep learning models on standard laboratory hardware—such as the NVIDIA T400 (4 GB VRAM) used in this study—imposes real constraints on batch size, training time, and the number of candidate architectures that can be practically evaluated. With more limited hardware, training times could increase dramatically, making the exploration of multiple architectures and the application of strategies such as SHA computationally prohibitive. In this sense, reporting parameters, FLOPs, and memory requirements is not merely an academic exercise but a practical guide for research groups seeking to replicate or adapt this methodology within the resource constraints typical of biomedical laboratory environments. Inference latency, while not critical for real-time monitoring given that bioreactor images are acquired at intervals of several minutes, provides an additional reference for deployment feasibility. A detailed discussion of these considerations is provided in Section 3.6.

According to the official Ultralytics documentation, YOLOv8 detection models range from approximately 3.2 million parameters and 8.7 GFLOPs for the nano detection variant to around 68.2 million parameters and 257.8 GFLOPs for the extra-large variant at an input resolution of 640 × 640 pixels (Ultralytics YOLO11, 2025; YOLOv8 Architecture, 2025). For YOLOv11, Ultralytics reports further gains in parameter efficiency. The YOLOv11 detection family spans from 2.6 M parameters and 6.5 GFLOPs for the nano model to 56.9 M parameters and 194.9 GFLOPs for the extra-large model at 640 × 640 resolution. Thus, YOLOv11 achieves comparable or superior accuracy to YOLOv8 while requiring fewer parameters and FLOPs, particularly in the medium and large variants, highlighting its efficiency-oriented refinements.

RT-DETR exhibits a different complex profile. In its small configuration, the model contains approximately 21 million parameters, with FLOPs that vary depending on the number of decoder layers used (Zhang et al., 2025). While larger than YOLO-nano models, RT-DETR benefits from end-to-end design and parallelizable transformer blocks, enabling competitive accuracy with relatively moderate latency compared to conventional DETR variants.

From a biomedical perspective, where computational resources may be limited and real-time feedback is often desirable, these differences are critical. Small and medium YOLO variants may offer the best compromise for routine use in laboratory settings, while larger variants can serve as upper-bound references for accuracy in research contexts. Reporting both parameter counts and FLOPs provides transparency, enabling other groups to select the most appropriate trade-off for their specific experimental environment.

#### 2.3.4 Pretrained initialization

All models in this study were initialized with weights pretrained on the COCO dataset (Lin et al., 2014), a large-scale benchmark that contains over 200,000 labeled images across 80 object categories. Transfer learning from COCO has become standard practice in biomedical computer vision, as it provides networks with robust low- and mid-level visual features such as edge detectors, texture representations, and shape encoders, which are transferable across domains (Plested et al., 2025). This initialization accelerates convergence during training, reduces the risk of overfitting on relatively small biomedical datasets, and often leads to significant improvements in generalization performance.

However, transfer learning from natural image datasets such as COCO is not without limitations when applied to biomedical microscopy images. The domain gap between natural photographs and fluorescence microscopy images is substantial: COCO images contain diverse real-world scenes with complex backgrounds and multi-scale objects, whereas microscopy images exhibit uniform backgrounds, domain-specific staining patterns, and objects with highly regular morphology. This domain gap may result in suboptimal feature representations in early training stages, potentially requiring more epochs to adapt pretrained features to the target domain. Additionally, COCO does not contain any microscopy or biological imagery, meaning that higher-level semantic features may transfer less effectively than low-level ones. These limitations were partially mitigated in this study through the use of domain-specific data augmentation strategies and extended training budgets, as detailed in Sections 2.4.3, 2.4.4.

In YOLOv8 and YOLOv11, pretrained weights are released by Ultralytics for all model sizes (n, s, m, l, x), making them directly accessible through the official framework. Similarly, the RT-DETR implementation provides pretrained weights on COCO, which were used as initialization for our transformer-based experiments.

#### 2.3.5 Implementation details

All detection models were implemented using the Ultralytics framework (version 8.3.225), which provides unified training and inference pipelines for YOLOv8 and YOLOv11 architectures. RT-DETR models were trained using the same framework, ensuring consistent data loading, augmentation, and evaluation procedures across all experiments.

Training was conducted on an NVIDIA T400 GPU with 4 GB of video memory. Due to memory constraints, a batch size of 2 was used uniformly for all models. Mixed-precision training (FP16) was enabled via PyTorch’s automatic mixed precision (AMP) to reduce memory footprint and accelerate computation. The software environment consisted of Python 3.10.19, PyTorch 2.6.0, and CUDA 12.4. All experiments were executed in deterministic mode with a fixed random seed (seed = 0) to ensure reproducibility across runs (Table 1).

**TABLE 1:** Software and hardware configuration.

Component	Specification
GPU	NVIDIA T400 (4 GB VRAM)
Deep learning framework	PyTorch 2.6.0
CUDA version	12.4
Training framework	Ultralytics 8.3.225
Python version	3.10.19
Precision	FP16 (automatic mixed precision)
Random seed	0 (deterministic mode enabled)

#### 2.3.6 Rationale for model selection and evaluation scope

The selection of models in this study was guided by the need to balance methodological diversity, state-of-the-art relevance, and practical feasibility for biomedical applications. YOLOv8 was included as a consolidated baseline, having rapidly become one of the most widely adopted frameworks in both industrial and academic settings since its release in 2023. Its anchor-free head and decoupled classification and regression branches made it an appropriate reference point for object detection in microscopy data.

YOLOv11, released by Ultralytics in 2024, was incorporated as the most recent official evolution of the YOLO family. The official Ultralytics documentation reports consistent gains in accuracy and computational efficiency compared to YOLOv8. Including YOLOv11 allowed us to assess whether these improvements generalize to the biomedical domain, providing an opportunity to evaluate the impact of ongoing architectural refinements on a task substantially different from the natural images in which these models are typically benchmarked.

We also considered it essential to evaluate a non-CNN alternative. For this reason, RT-DETR was included as a transformer-based detector, representing a different architectural paradigm in which end-to-end object detection is achieved without the need for non-maximum suppression. This comparison was intended to test whether transformer-based models could offer advantages in scenarios such as microcarrier detection, where objects are numerous, clustered, and exhibit variable fluorescence intensity.

Segmentation-based approaches, including Segment Anything Model (SAM), were also considered during the design phase of this study. A preliminary proof-of-concept evaluation indicated that while SAM demonstrated reasonable microcarrier detection capabilities, it lacked the ability to reliably classify detected objects into colonized and non-colonized categories—a critical requirement for colonization quantification. This limitation, inherent to the class-agnostic nature of SAM, motivated the adoption of end-to-end object detection models that jointly perform localization and appearance-based classification in a single framework.

Prior to adopting a deep learning-based framework, classical image processing approaches were also explored. In particular, fluorescence intensity thresholding was evaluated as a straightforward method to classify microcarriers based on the presence or absence of internal green signal. However, this approach proved unreliable due to the inherent variability in fluorescence intensity across images: highly colonized samples exhibit strong fluorescence signals that make it difficult to correctly identify empty microcarriers when the threshold is set high, while images with low colonization present weak signals that lead to misclassification of colonized carriers when the threshold is lowered. This sensitivity to threshold selection, combined with variability across acquisition sessions and culture days, made classical thresholding insufficient for robust and reproducible colonization quantification.

It is important to clarify that YOLOv10, while sometimes cited in literature, was not considered in this study. Unlike YOLOv8 and YOLOv11, YOLOv10 is not an official Ultralytics release, but rather an external development that focuses primarily on energy-efficient deployment and NMS-free training. For the sake of coherence, and to avoid introducing architectures with divergent goals, we restricted our analysis to the official Ultralytics lineage, complemented by RT-DETR as an external reference. To the best of our knowledge, no versions beyond YOLOv11 have been officially released by Ultralytics at the time of writing.

### 2.4 Training protocol

#### 2.4.1 Training setup

All experiments were conducted at a fixed input resolution of 640 × 640 pixels to standardize feature-map dimensions across architectures and ensure fair comparison. A batch size of 2 was used uniformly for all models, constrained by GPU memory limitations (4 GB VRAM).

The AdamW optimizer was employed with an initial learning rate of 1 × 10^−3^, weight decay of 5 × 10^−4^, and cosine annealing schedule with a 3-epoch linear warmup (Loshchilov and Hutter, 2017). The learning rate decayed to 1% of its initial value by the end of training. All models were initialized from COCO-pretrained weights to leverage learned low-level features and accelerate convergence on the relatively small microcarrier dataset. Table 2 summarizes the complete training configuration.

**TABLE 2:** Training hyperparameters and experimental setup.

Parameter	Value
Image size	640 × 640 px
Batch size	2
Optimizer	AdamW
Initial learning rate	1 × 10^−3^
Weight decay	5 × 10^−4^
Final learning rate	1 × 10^−5^ (1% of initial)
Warmup	3 epochs (linear)
Precision	FP16 (mixed)
LR schedule	Cosine annealing

#### 2.4.2 Optimization strategy

Rather than training all configurations for a fixed and uniform number of epochs, we employed a multi-stage model selection strategy based on the Successive Halving Algorithm (SHA) (X. Zhang and Duh, 2024). SHA is a principled early-stopping method that allocates computational resources progressively: all candidate models are trained for an initial budget, evaluated on a validation metric, and only the top-performing subset is promoted to the next stage with an increased training budget. This process repeats until a small set of finalists remains. Unlike its asynchronous variant ASHA (Wojciuk et al., 2024), our implementation operates synchronously all models complete each stage before promotion decisions are made.

The key methodological rationale behind SHA is not to maximize the individual performance of each candidate model, but rather to efficiently identify the best-performing architecture under controlled and comparable conditions. By keeping all hyperparameters fixed across models and varying only the training budget—the number of epochs, which we consider the most critical variable in this context—SHA ensures that model comparisons are fair and reproducible. This approach is particularly well suited to biomedical research settings where exhaustive training and individual hyperparameter tuning of every candidate architecture would require computational resources beyond those typically available in a laboratory environment.

Twelve candidate architectures entered Stage 1.

- YOLOv8: n, s, m, l, x (5 models)
- YOLOv11: n, s, m, l, x (5 models)
- RT-DETR: l, x (2 models)

All models were trained from COCO-pretrained initialization for 20 epochs in Stage 1. Models were ranked by mAP_50_–_95_ on the validation set, and the top 4 were promoted to Stage 2. In Stage 2, these four models were retrained from scratch (again from COCO-pretrained weights) for 50 epochs, and the top 2 were selected for the final stage. In Stage 3, the two finalists were retrained from scratch for 150 epochs, with YOLOv8-l emerging as the best-performing model.

This staged retraining approach where each stage restarts from pretrained initialization rather than continuing from previous checkpoints ensures that promoted models benefit from the full training dynamics (including learning rate warmup, cosine annealing schedule, and augmentation policies) at each budget level, rather than inheriting potentially suboptimal optimization trajectories from shorter runs. The progressive elimination strategy reduced total computational cost by approximately 60% compared to exhaustively training all 12 models for 150 epochs, while maintaining high confidence in identifying the optimal architecture for the microcarrier detection task.

#### 2.4.3 Validation and checkpointing

Throughout training, model performance was monitored on the hold-out validation set (140 images, 15% of the dataset) at the end of each epoch. The best checkpoint for each model was selected based on validation mAP_50_–_95_, and only these checkpoints were used for subsequent evaluation and comparison.

Post-training threshold calibration was conducted using the validation set exclusively. Confidence and IoU thresholds were optimized via grid search to minimize the Mean Absolute Error (MAE) of colonization percentage estimation. A total of 44 threshold combinations were evaluated, and the optimal configuration (confidence = 0.55, IoU = 0.4) was selected based on validation performance. This calibrated configuration was then applied uniformly during test set evaluation.

#### 2.4.4 Data augmentation

A consistent augmentation pipeline was applied across all models to ensure comparability. The pipeline included (Cubuk et al., 2019; Wang et al., 2022; Zhong et al., 2017):

- Horizontal flip (probability = 0.5): Mirrors images along the vertical axis to increase geometric diversity.
- Rotation (±15°): Random rotation within the specified range.
- Scale (factor = 0.8): Random downscaling to simulate distance variation in microscopy imaging.
- HSV perturbation: Random adjustments to hue (±1.5%), saturation (±70%), and value (±40%) to improve robustness against illumination and staining variability.
- Mosaic augmentation: Combines four training images into a single composite, increasing contextual diversity and effective batch size. Mosaic was disabled during the final 10 epochs (close_mosaic = 10) to allow the model to fine-tune on unaltered images before convergence.
- Random erasing (probability = 0.4): Randomly masks rectangular image regions to encourage robustness to partial occlusion.
- RandAugment: Automated augmentation policy that applies a sequence of randomly selected transformations with tuned magnitudes.

These augmentations were implemented through the Ultralytics framework’s native pipeline, ensuring identical preprocessing across YOLOv8, YOLOv11, and RT-DETR models. Augmentation parameters were held constant across all experiments; no model-specific tuning was performed. This design choice prioritizes fair architectural comparison over maximizing individual model performance. Importantly, all augmentation procedures were applied exclusively to the training subset after dataset splitting, ensuring that validation and test sets remained unmodified and free from any form of data leakage.

#### 2.4.5 Reproducibility

To ensure reproducibility to the extent permitted by the proprietary constraints of this study, random seeds were fixed across all libraries (NumPy, PyTorch, Python) throughout the experiments, and deterministic mode was enabled in PyTorch. All training configurations, hyperparameters, data splits, augmentation parameters, and evaluation procedures are reported in full detail in this manuscript. All model architectures evaluated in this study are publicly available through the Ultralytics framework (version 8.3.225), and COCO-pretrained weights are freely accessible. The complete software and hardware environment is detailed in Table 1.

An anonymized version of the analysis code, including all library dependencies, modifications to the original framework, and implementation details of the image processing and annotation pipelines, is publicly available at Automated-Microcarrier-Bioreactor-detection/docs/METHODOLOGY.md at main manutfds/Automated-Microcarrier-Bioreactor-detection · GitHub. While the fluorescence microscopy images and trained model weights cannot be made publicly available due to proprietary restrictions, we encourage interested researchers to contact the corresponding authors to explore potential academic collaborations.

### 2.5 Inference and evaluation

#### 2.5.1 Inference pipeline

The inference workflow was designed to standardize post-processing across all detection models. All trained models (YOLOv8, YOLOv11, and RT-DETR) were evaluated at an input resolution of 640 × 640 pixels using mixed-precision inference (FP16) on the same GPU hardware employed during training (NVIDIA T400, 4 GB) to ensure fair latency and throughput comparison.

For all detection models, the output consisted of bounding boxes with associated confidence scores and class labels (FULL or EMPTY). Non-maximum suppression (NMS) was applied with an IoU threshold of 0.4 to remove redundant overlapping detections. Confidence thresholds were set according to the calibrated operating point (confidence = 0.55) determined during validation. Microcarriers were counted per image, and the proportion labeled as FULL defined the predicted colonization percentage (Equation 1):

$$Colonization\;\left(\%\right)=\frac{N_{Full}}{N_{Full}+N_{Empty}}x100$$ (1)

Inference latency per image (milliseconds) and throughput (frames per second, FPS) were also recorded as practical performance indicators, given their importance in real-time laboratory monitoring (Bochkovskiy et al., 2020).

#### 2.5.2 Evaluation metrics and performance indicators

Model performance was evaluated through three complementary categories: (i) image-level colonization accuracy, (ii) object-level detection accuracy, and (iii) computational efficiency.

##### 2.5.2.1 Image-level colonization accuracy

The primary evaluation criterion was the Mean Absolute Error (MAE) (Equation 2) between predicted and ground-truth colonization percentages:

$$MAE=\frac{1}{N}\sum_{i=1}^{N}\left(y_{i}-{ŷ}_{i}\right)$$ (2)

where $y_{i}$ and ${\hat{y}}_{i}$ denote ground-truth and predicted colonization percentages, respectively. MAE provides a directly interpretable measure of error in the same units (percentage points) used by laboratory technicians.

To complement MAE, the Mean Absolute Percentage Error (MAPE) (Equation 3) was also computed:

$$MAPE=\frac{100}{N}\sum_{i=1}^{N}\left|\frac{y_{i}-{ŷ}_{i}}{y_{i}}\right|$$ (3)

However, MAPE is sensitive to small denominators and can yield inflated values for images with low colonization; thus, MAE was prioritized as the primary colonization metric.

Additionally, the coefficient of determination (*R* ^2^) and systematic bias were reported to characterize the agreement between automated and manual quantifications. Bias was computed as the mean signed error (predicted − ground truth), with negative values indicating systematic underestimation.

##### 2.5.2.2 Object-level detection accuracy

Detection quality was assessed using mean Average Precision (mAP) at IoU thresholds of 0.50 and 0.50–0.95, following the COCO evaluation protocol (Lin et al., 2014).

- mAP_50_: Measures accuracy at a moderate overlap threshold (IoU ≥0.5), commonly used for object detection benchmarks.
- mAP_50_–_95_: Averages precision across IoU thresholds from 0.50 to 0.95 in steps of 0.05, providing a more comprehensive assessment of localization quality.

Additionally, the classical metrics Precision, Recall, and F1-score (Equation 4) were computed for each class (FULL, EMPTY):

$$P=\frac{\text{TP}}{\text{TP}+FP^{´}\;};R=\frac{\text{TP}}{\text{TP}+FN^{´}\;};\;F1=\frac{2\mathrm{PR}}{P+R\;}$$ (4)

Where TP, FP, and FN denote true positives, false positives, and false negatives. Precision quantifies how many detected microcarriers were correctly identified, while Recall measures what fraction of true microcarriers were successfully detected. The F1-score balances both metrics, summarizing the trade-off between over-detection and under-detection.

##### 2.5.2.3 Computational efficiency

Each model was characterized by.

- Inference latency: Time to process a single image (ms).
- Throughput: Number of images processed per second (FPS).
- Model size: Number of trainable parameters.
- Computational cost: Floating-point operations per image (GFLOPs).

These indicators are critical for future deployment in laboratory environments where rapid feedback could enable real-time bioprocess monitoring and adaptive bioprocess control.

### 2.6 Error analysis

A systematic error analysis was performed to identify the main factors contributing to deviations between automated and manual quantifications. The analysis focused on three complementary levels.

- Object level: False positives (FP) and false negatives (FN) were categorized by their likely causes, including: (a) microcarrier aggregates where overlapping carriers were detected as single objects or missed entirely, (b) low fluorescence intensity making colonized carriers difficult to distinguish from empty ones, (c) edge effects where partially visible microcarriers at image borders were inconsistently detected, and (d) debris or artifacts misclassified as microcarriers.
- Image level: The distribution of prediction errors was analyzed to identify systematic patterns. Images with high colonization (>80%) and low colonization (<20%) were examined separately to assess whether the model exhibited range-dependent bias. Bland-Altman analysis was used to visualize agreement between predicted and ground-truth colonization across the full range of values.
- Dataset level: Variability in model performance was examined across different experimental conditions present in the dataset, including variations in background intensity, days of culture, cell confluence levels, and microscopy acquisition parameters.

## 3 Results

### 3.1 Exploratory data analysis (EDA)

**FIGURE 2:**
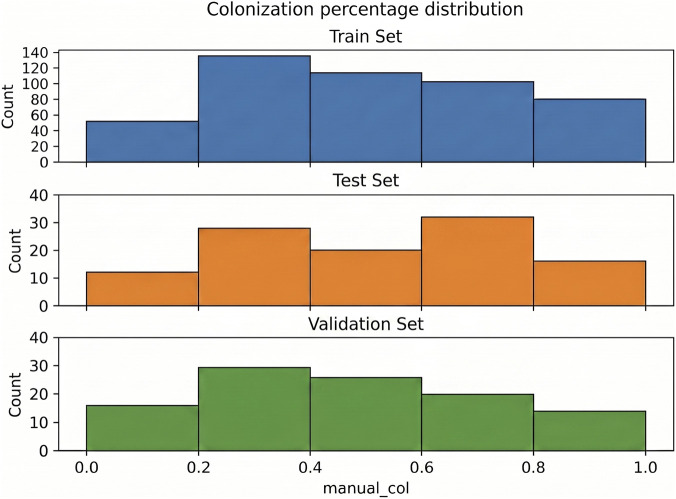
Colonization percentage distribution across dataset splits. Histogram showing the distribution of colonization percentages (manual_col) for the training, validation, and test subsets. The three splits display highly similar interval profiles across the 0–1 range, indicating consistent annotation behaviour and uniform colonization-level representation throughout the dataset.

The analyzed dataset (n = 699 images) contained 46,982 annotated objects with an almost perfectly balanced class distribution (50.18% “Empty”, 49.82% “Full”), minimizing majority-class bias and supporting consistent model evaluation. After preprocessing, the data were split into training, validation, and test subsets (70%/15%/15%), comprising 32,862, 7,056, and 7,057 objects, respectively. Annotation-quality assessments showed that colonization percentages followed nearly identical distributions across all splits indicating uniform labeling criteria and ruling out split-dependent annotation bias (Figure 2).

**FIGURE 3:**
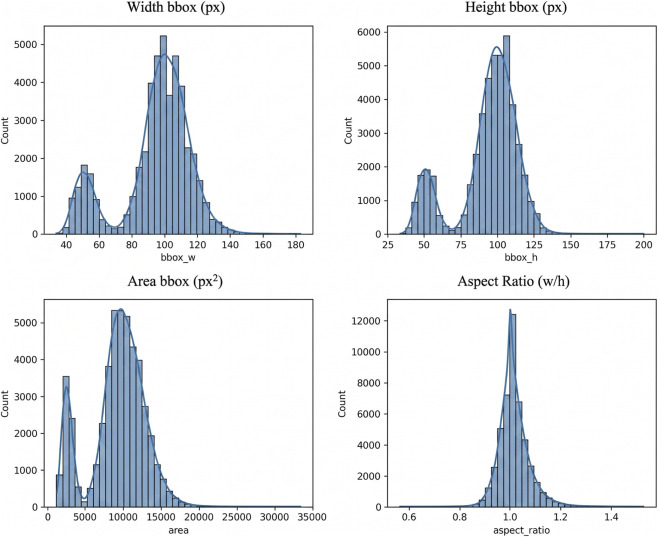
Histogram distributions of bounding-box geometry. Width, height, and area show two compact size modes, while the aspect ratio is tightly centered around 1.0, indicating highly uniform, near-circular object morphology across the dataset.

Geometric analyses further revealed highly regular object morphology: width, height, and area histograms exhibited two compact size modes, while aspect ratios were sharply centered around 1.0, consistent with near-circular object geometry. This homogeneity confirms both labeling stability and structural consistency across the dataset, supporting reliable detector training and anchor-box configurations aligned with the observed object shapes (Figure 3). Notably, no meaningful geometric differences were found between FULL and EMPTY classes, confirming that colonization status cannot be inferred from object morphology alone and further justifying the use of an appearance-based classification approach.

### 3.2 Model selection via successive halving

#### 3.2.1 Stage 1: initial screening (20 epochs)

Following an SHA screening strategy with a reduction factor ETA = 3 we conducted a first-stage evaluation of 12 candidate object-detection models. All models were trained from COCO-pretrained weights for 20 epochs and ranked by validation mAP_50–95_. First-stage performance spanned a wide range (0.219–0.577), with YOLOv8-l achieving the best accuracy (mAP_50–95_ = 0.577; 3.223 h), followed by YOLOv8-x (0.568; 10.133 h), YOLOv11-l (0.566; 2.711 h), and YOLOv11-x (0.566; 10.298 h), while YOLOv11-m showed comparable performance (0.562; 2.251 h). In contrast, the RETDR variants underperformed markedly (0.241 and 0.219) despite substantially longer training times (4.416–12.484 h) (Table 3). Consistent with ETA = 3, SHA promoted the top four models from this rung (reducing the pool from 12 to 4) for subsequent stages with increased training budget, prioritizing the strongest accuracy candidates while controlling computational cost.

**TABLE 3:** Stage-1 performance screening of candidate models under the SHA early-ranking scheme.

Model	Epoch	Time (h)	mAP_50–95_
YoloV8 n	20	0.455	0.489
YoloV8 s	20	1.020	0.545
YoloV8 m	20	2.178	0.548
YoloV8 l	**20**	**3.223**	**0.577**
YoloV8 x	**20**	**10.133**	**0.568**
YoloV11 n	20	0.512	0.488
YoloV11 s	20	1.066	0.531
YoloV11 m	20	2.251	0.562
YoloV11 l	**20**	**2.711**	**0.566**
YoloV11 x	**20**	**10.298**	**0.563**
RETDR-l	20	4.416	0.241
RETDR-x	20	12.484	0.219

Bold values indicate the best-performing models advancing to the next stage.

#### 3.2.2 Stage 2: Intermediate evaluation (50 epochs)

In the second SHA stage, the four candidates promoted from the initial pool of 12 models (selected at 20 epochs) were retrained with an increased budget of 50 epochs to refine the ranking under a higher-fidelity evaluation. All promoted models showed consistent gains in mAP_50_–_95_ compared with Stage 1, confirming that the early-stage selection retained the most promising configurations: YOLOv8-l improved from 0.577 to 0.655 (7.951 h), YOLOv11-l from 0.566 to 0.641 (6.694 h), YOLOv8-x from 0.568 to 0.637 (24.883 h), and YOLOv11-x from 0.563 to 0.631 (26.004 h) (Table 4). Notably, while YOLOv8-l remained the top-performing model in absolute accuracy, the ranking partially shifted relative to Stage 1, with YOLOv11-l surpassing YOLOv8-x at 50 epochs, suggesting improved competitiveness under extended training. From a computational standpoint, the “x” variants incurred substantially longer training times (≈25–26 h) for comparatively modest accuracy advantages, whereas the “l” variants achieved a more favorable accuracy–cost trade-off, supporting their prioritization for subsequent stages where ASHA allocates progressively larger budgets to an increasingly smaller set of models (Table 4).

**TABLE 4:** Stage-2 performance (50 epochs). Top 2 models promoted to Stage 3**.**

Model	Epoch	Time (h)	mAP_50–95_	Delta mAP	Time factor
YoloV8 l	50	7.951	0.655	+0.078	2.47x
YoloV8 x	50	24.883	0.637	+0.069	2.46x
YoloV11 l	**50**	**6.694**	**0.641**	**+0.075**	**2.47x**
YoloV11 x	50	26.004	0.631	+0.068	2.53x

Bold values indicate the best-performing models advancing to the next stage.

#### 3.2.3 Stage 3: Final evaluation (150 epochs)

**FIGURE 4:**
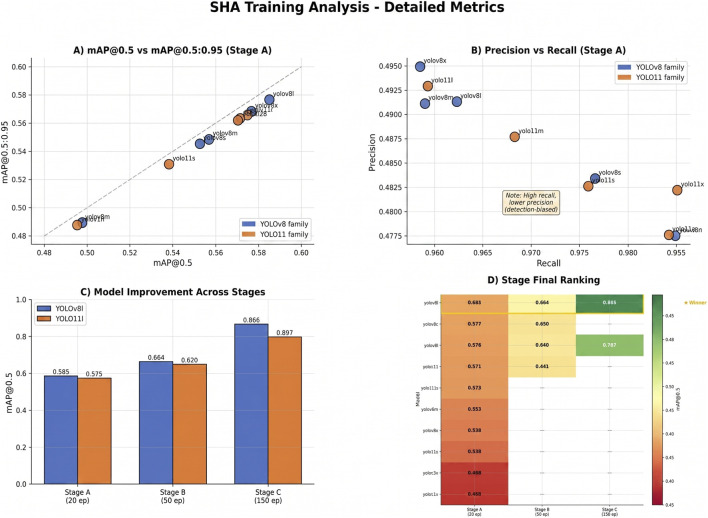
SHA-based model selection analysis. **(A)** Scatter plot of mAP_50_ (mAP@0.5) vs. mAP_50_–_95_ (mAP@0.5:0.95) at Stage 1, showing consistent correlation between metrics across model families. **(B)** Precision-recall trade-off at Stage 1, demonstrating high-recall operating regime for all YOLO models. **(C)** Performance improvement across SHA stages for the two finalists, highlighting the widening gap between YOLOv8-l and YOLOv11-l under extended training. **(D)** Model performance comparison across SHA stages (20, 50, and 150 epochs). The heatmap illustrates the progressive filtering of candidate models based on mAP_50_, with eliminated models indicated by dashes and the best-performing architecture highlighted. The visualization summarizes the early stopping–driven ranking process and the selection of the final model configuration.

In the third SHA, the search was further narrowed to the two leading candidates from Stage 2 and evaluated under a substantially increased training budget of 150 epochs. Both models continued to benefit from additional training, indicating that their earlier performance was stable under extended optimization: YOLOv8-l improved from mAP_50_–_95_ of 0.655 at 50 epochs to 0.856 at 150 epochs (24.302 h), while YOLOv11-l increased from 0.641 to 0.790 (20.160 h) (Table 5). Importantly, the higher-budget evaluation accentuated the performance gap between the finalists, with YOLOv8-l expanding its lead from 0.014 at 50 epochs to 0.066 at 150 epochs, suggesting superior scaling with prolonged training in this setting. Training times increased approximately linearly with the epoch budget, consistent with the expected computational behavior as SHA allocates progressively larger resources to progressively fewer models. Overall, Stage 3 confirms YOLOv8-l as the best-performing configuration under the highest training budget considered, supporting its selection for subsequent experimentation and/or final deployment (Table 5; Figure 4).

**TABLE 5:** Stage-3 final performance.

Model	Epoch	Time (h)	mAP_50–95_	Delta mAP	Time factor
YoloV8 l	**150**	**24.302**	**0.856**	**+0.201**	**3.06x**
YoloV11 l	150	20.160	0.790	+0.149	3.01x

Bold values indicate the best-performing models advancing to the next stage.

### 3.3 Training dynamics of the best model

**FIGURE 5:**
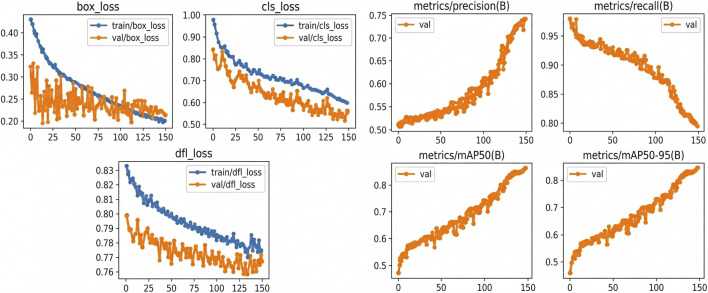
Training and validation dynamics of loss functions and performance metrics. Evolution of training and validation losses (box, classification, and DFL), together with validation precision, recall, and mAP metrics over epochs. The curves show stable and progressive optimization, with decreasing loss terms and consistently improving performance metrics, indicating effective convergence and absence of overfitting across the monitored training process.

Regarding the best model, the learning curves over the full training budget (up to 150 epochs) indicate stable optimization and sustained improvements in detection performance. Training losses for localization and classification (box, cls, and DFL) decrease consistently throughout training, suggesting progressive refinement of both bounding-box regression and class discrimination. Importantly, validation losses follow a compatible downward trend, despite higher variance in the validation box loss, supporting good generalization and providing no clear evidence of late-stage overfitting. In parallel, the detection metrics improve steadily, with both mAP_50_ and the stricter mAP_50_–_95_ increasing almost monotonically and reaching their best values at the end of training, consistent with the final high-budget performance observed in Stage 3. A notable trade-off is observed between precision and recall: precision rises markedly across epochs, while recall gradually decreases, suggesting that the model becomes increasingly conservative by prioritizing higher-confidence predictions (reducing false positives) at the expense of missing some instances at the default operating point. Overall, these trends confirm that extended training yields meaningful gains in accuracy and localization quality, while highlighting the importance of selecting an appropriate inference threshold to balance precision and recall for the intended application (Figure 5).

After model training, we first reported standard detection metrics on the validation split (i.e., Precision = 0.762, Recall = 0.855, mAP_50_ = 0.866, and mAP_50_–_95_ = 0.856), which confirmed stable convergence and strong localization performance under increasingly strict IoU criteria.

### 3.4 Test set performance and colonization estimation

#### 3.4.1 Detection metrics on test set

**FIGURE 6:**
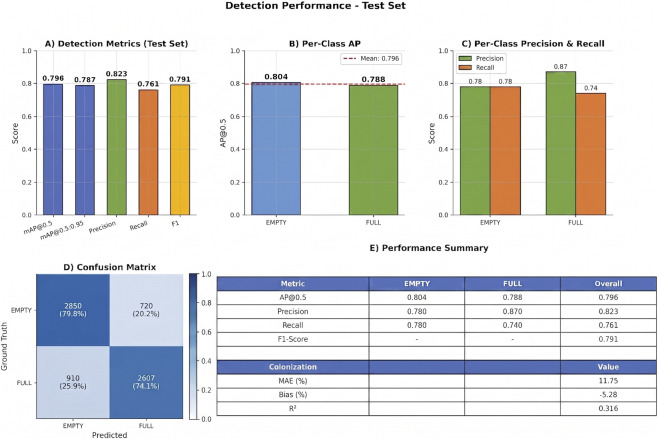
Comprehensive detection performance on the test set. Summary of model performance on the test set. Panel **(A)** shows global detection metrics (mAP_50_ = mAP@0.5, mAP_50_–_95_ = mAP@0.5&ndash;0.95, precision, recall, F1). Panel **(B)** presents per-class AP values, while Panel **(C)** compares per-class precision and recall. Panel **(D)** displays the confusion matrix with class-wise accuracy distribution. Panel **(E)** consolidates overall performance metrics, including colonization-related regression indicators (MAE, bias, and *R* ^2^). Together, these results indicate balanced behaviour across classes and robust detection performance under real test-set conditions.

Because our downstream objective is not only accurate detection but also accurate colonization quantification (i.e., the percentage of colonized microcarriers), we subsequently performed threshold calibration on the validation set. This calibration step does not modify the trained model weights; instead, it adjusts post-processing decision thresholds to align the detector’s operating point with the requirements of the application (analogous to tuning the sensitivity of a sensor). Specifically, we evaluated 44 confidence/IoU combinations via grid search and selected the configuration that minimized colonization error, yielding an optimal setting of confidence = 0.55 and IoU = 0.4, with a validation MAE = 10.71%. Here, the confidence threshold controls how certain the model must be to accept a detection (lower values increase false positives; higher values increase missed detections), while MAE measures the average absolute deviation (in percentage points) between predicted and ground-truth colonization per image. Under this calibrated operating point, the model achieved an R2 = 0.37, indicating a moderate linear association: the model captures the overall trend in colonization across images, but with noticeable variability at the individual-image level (Figure 6).

Detection performance on the held-out test set remained robust (though lower than validation), with Precision = 82.3% and Recall = 76.2%, indicating a relatively conservative operating regime, i.e., a low false-positive rate at the cost of missing approximately 24% of true objects, consistent with mAP_50_ = 0.796 and mAP_50_–_95_ = 0.787; importantly, this operating point is aligned with the downstream colonization-estimation objective after calibration, but it can also partially explain a tendency to under-count colonized (“FULL”) instances when detections are missed.

#### 3.4.2 Colonization estimation accuracy

**FIGURE 7:**
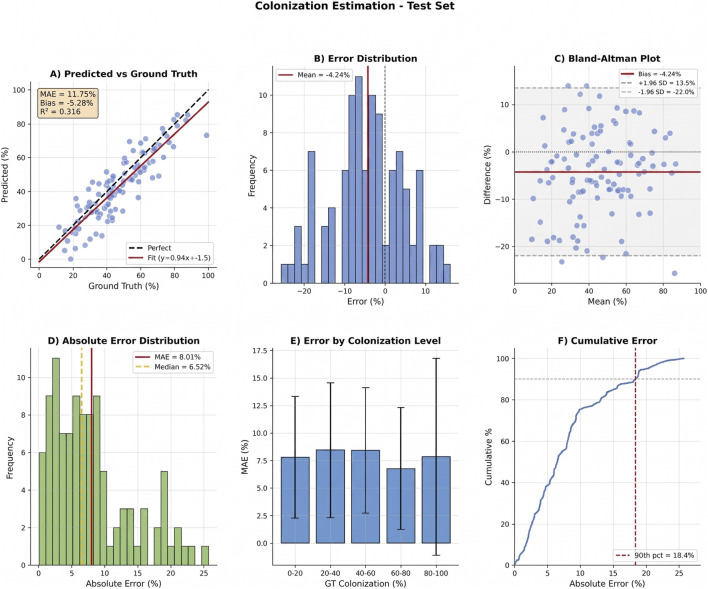
Colonization estimation performance on the test set. Comprehensive evaluation of colonization percentage estimation on the test set. Panel **(A)** shows predicted versus ground truth values with regression fit and global error metrics (MAE, bias, *R* ^2^). Panel **(B)** illustrates the distribution of percentage error, while Panel **(C)** presents a Bland–Altman analysis of agreement. Panel **(D)** displays the absolute error distribution, and Panel **(E)** reports mean absolute error across discrete colonization levels. Panel **(F)** shows the cumulative absolute error, indicating the proportion of samples below a given error threshold. Together, these plots characterize both systematic and random error components in model predictions.

The ultimate evaluation criterion for this application is the accuracy of colonization percentage estimation, the proportion of microcarriers classified as FULL in each image. On the test set, the model achieved a Mean Absolute Error (MAE) of 11.75 percentage points, meaning predictions deviated on average by approximately ±12% from ground-truth colonization values (Figure 7A).

Agreement analysis revealed moderate correspondence with manual quantification (*R* ^2^ = 0.316), with a regression fit of Predicted = 0.94 × Ground Truth −1.5. This slightly compressed response, combined with a systematic underestimation bias of −5.28% (mean signed error), indicates that the model tends to predict colonization values slightly lower than the true values. The bias is likely attributable to the reduced recall at the calibrated operating point, which preferentially misses detections rather than creating false positives. Additionally, the inherent visual heterogeneity of colonized microcarriers may contribute to this bias: FULL microcarriers can display a wide range of fluorescence intensities and spatial distributions depending on cell coverage degree, culture day, and donor variability, making the FULL/EMPTY decision boundary inherently ambiguous for borderline cases. This is further supported by the error analysis results (see Section 3.5), which show that FULL→EMPTY misclassifications and false negatives increase progressively with colonization level, suggesting that densely colonized images—where overlapping fluorescence signals and complex cell distributions are more frequent—pose a greater classification challenge. The most promising strategy to mitigate this bias in future work would be to expand the training dataset with a greater number and diversity of FULL microcarrier examples, particularly those exhibiting weak, partial, or heterogeneous fluorescence patterns. A richer representation of borderline colonization cases would allow the model to better learn the decision boundary between classes, potentially reducing the systematic underestimation observed in this study.

Bland-Altman analysis (Figure 7C) provided complementary insight into agreement across the colonization range. The limits of agreement (approximately −22% to +13.5%) indicate that while most predictions fall within an acceptable range, a minority of challenging samples drive larger deviations. The asymmetric limits (wider on the negative side) are consistent with the underestimation bias.

Error distribution analysis (Figure 7B–D) showed that the absolute error distribution was right-skewed, with median absolute error of 6.52% and 90th percentile of 18.4%. This indicates that approximately 67% of images are estimated with good accuracy (<10% absolute error), while a tail of difficult cases exhibits larger errors (Figure 7F). The Mean Absolute Percentage Error (MAPE) of 27.92% appears high but is inflated by images with low ground-truth colonization (<20%), where even small absolute errors translate to large relative errors, a known artifact of percentage-based metrics.

Stratified analysis by colonization level (Figure 7E) revealed that errors were not uniformly distributed: images with intermediate colonization (40%–60%) tended to show higher MAE than those at extreme values (<20% or >80%). This pattern likely reflects the increased difficulty of distinguishing FULL from EMPTY microcarriers when both classes are similarly represented and the classification boundary is most challenged.

From a practical standpoint, the cumulative error distribution (Figure 7F) indicates that approximately 70% of test images were estimated with <15% absolute error, and approximately 85% with <20% error. For routine laboratory monitoring where the goal is to track colonization trends rather than achieve exact quantification, this accuracy level represents a substantial improvement over the inter-operator variability typically observed in manual counting (Cai et al., 2024; Salinas et al., 1997).

### 3.5 Error analysis

To understand the sources of prediction errors and identify opportunities for improvement, we conducted a systematic analysis of detection outcomes on the test set. Detections were categorized as true positives (TP), false positives (FP), false negatives (FN), or misclassifications (correct localization but incorrect class assignment) based on IoU matching with ground-truth annotations at the calibrated threshold (IoU = 0.4). This analysis employs a four-category classification scheme that separates misclassifications (correct localization, incorrect class) from pure detection errors, providing complementary insight to the standard precision-recall metrics reported.

#### 3.5.1 Distribution of detection errors

**FIGURE 8:**
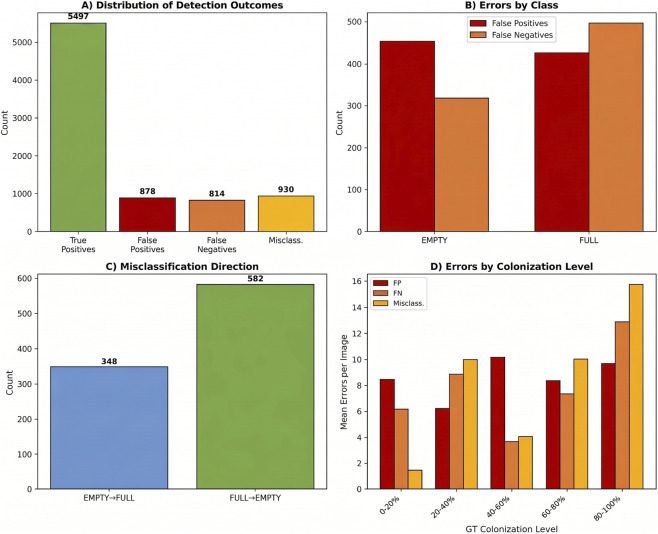
Distribution and characterization of detection errors on the test set. **(A)** Overall distribution of detection outcomes showing true positives (TP = 5,497), false positives (FP = 878), false negatives (FN = 814), and misclassifications (930). **(B)** Class-specific error distribution. False positives were slightly more frequent for the EMPTY class, while false negatives were elevated for the FULL class. **(C)** Directionality of misclassification errors. FULL→EMPTY misclassifications (582) substantially exceeded EMPTY→FULL errors (348), indicating a systematic tendency to underestimate colonization. **(D)** Mean errors per image stratified by ground-truth colonization level. False positive rates remained stable across colonization bins, while false negatives and misclassifications increased with colonization level, reflecting the greater challenge of classifying densely colonized samples.

Figure 8A summarizes the distribution of detection outcomes across the test set. Of 8,119 detection events analyzed, 5,497 (67.7%) were true positives, while the remaining errors were distributed among false positives (878, 10.8%), false negatives (814, 10.0%), and misclassifications (930, 11.5%). The relatively balanced distribution between FP and FN indicates that the calibrated operating point achieved a reasonable trade-off between over-detection and under-detection.

Error analysis by class (Figure 8B) revealed slightly higher false positive rates for the EMPTY class compared to FULL, while false negative rates were elevated for the FULL class. This asymmetry suggests that the model tends to miss some colonized microcarriers (detecting them as background) while occasionally misidentifying background regions or artifacts as empty microcarriers.

Misclassification analysis (Figure 8C) showed a pronounced directional bias: FULL→EMPTY misclassifications (582 instances) substantially outnumbered EMPTY→FULL errors (348 instances). This pattern indicates that the model more frequently fails to recognize colonization than it falsely attributes colonization to empty carriers. From a biological interpretation perspective, this is likely to reflect cases where colonized microcarriers exhibit weak or partial fluorescence signals that fall below the model’s learned decision boundary for the FULL class. This directional bias is consistent with the systematic underestimation of colonization percentage observed previously.

#### 3.5.2 Error distribution across colonization levels

The relationship between ground-truth colonization level and error frequency revealed important patterns (Figure 8D). False positive rates remained relatively stable across colonization bins (approximately 6–10 errors per image), suggesting that FP sources, such as debris, aggregates, or edge artifacts, are largely independent of the colonization state.

In contrast, both false negatives and misclassifications showed a clear positive correlation with colonization level. Images with high colonization (80%–100%) exhibited substantially more FN and misclassification errors (approximately 13–16 per image) compared to low-colonization images (0%–20%, approximately 1–6 errors per image). This pattern has two likely explanations: (i) highly colonized samples contain more FULL microcarriers, providing more opportunities for the dominant error mode (FULL→EMPTY misclassification); and (ii) densely colonized regions may present more challenging visual conditions, including overlapping fluorescence signals, touching microcarriers, and complex cell distributions that complicate both detection and classification.

These findings suggest that model performance could be improved through targeted strategies such as: (i) augmenting training data with more high-colonization examples; (ii) implementing class-balanced sampling during training; or (iii) developing specialized post-processing for high-density regions.

#### 3.5.3 Qualitative error analysis

**FIGURE 9:**
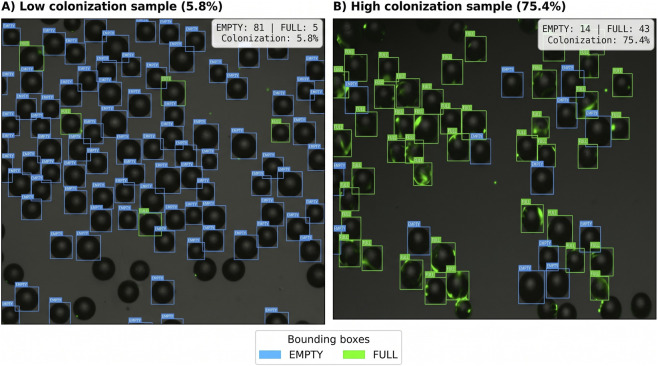
Representative detection results across the colonization spectrum. **(A)** Low-colonization sample (5.8% colonization). The model correctly identifies the predominance of empty microcarriers (blue boxes) with few colonized carriers (green boxes). Detection accuracy is high in sparse conditions with well-separated objects. **(B)** High-colonization sample (75.4% colonization). The model successfully detects most microcarriers but faces increased classification challenges. Colonized microcarriers with strong fluorescence (green boxes) are reliably identified, while some carriers with weak or partial fluorescence are misclassified as empty. Bounding boxes indicate detected microcarriers: blue = EMPTY, green = FULL. Detection results panel shows class counts and calculated colonization percentage.

Visual inspection of detection results across the colonization spectrum (Figure 9) provided additional insight into error sources.

In low-colonization images (Figure 9A 5.8% colonization), the model successfully detected the majority of microcarriers with correct class assignments. The predominance of EMPTY detections (blue boxes) accurately reflects the sample state. Errors in these images typically involved: (i) missed detections at image boundaries where microcarriers were partially visible; (ii) occasional false positives from debris or optical artifacts; and (iii) rare misclassifications of microcarriers with very faint autofluorescence.

In high-colonization images (Figure 9B 75.4% colonization), detection remained robust, but classification errors became more apparent. Several patterns were observed: (i) microcarriers with partial or asymmetric cell coverage were sometimes classified as EMPTY despite visible fluorescence; (ii) adjacent colonized microcarriers with overlapping fluorescence signals occasionally resulted in merged or missed detections; (iii) out-of-focus microcarriers in deeper focal planes showed reduced fluorescence intensity, leading to FULL→EMPTY misclassifications.

These qualitative observations are consistent with the quantitative findings and highlight that the primary challenge lies not in microcarrier detection *per se*, but in the binary classification of colonization status for borderline cases, particularly those with weak, partial, or obscured fluorescence signals.

#### 3.5.4 Implications for deployment

The error analysis has several practical implications. First, the systematic FULL→EMPTY bias suggests that colonization estimates should be interpreted as conservative lower bounds; true colonization may be slightly higher than reported values. Second, the increased error rates at high colonization levels indicate that model uncertainty is not uniform across operating conditions, a consideration for quality control protocols that might implement additional manual verification for samples exceeding certain colonization thresholds. Third, the relatively stable FP rate across conditions suggests that false alarms from artifacts do not scale with biological variability, supporting consistent performance across diverse sample states.

### 3.6 Computational efficiency

To assess the computational viability of the comparative evaluation framework, we benchmarked the efficiency of all 12 candidate architectures. Measurements were performed on the same hardware used for training (NVIDIA T400, 4 GB VRAM) at 640 × 640 input resolution with FP16 precision. Latency averaged over 50 iterations following 10 warmup runs to ensure stable measurements.

Table 6 summarizes the computational characteristics of the evaluated models. From a training perspective, computational efficiency is critical to making a multi-architecture comparative study feasible on standard laboratory hardware. YOLOv11 variants consistently exhibited lower parameter counts and GFLOPs than their YOLOv8 counterparts at equivalent model sizes (e.g., YOLOv11-l: 25.4 M parameters, 87.6 GFLOPs vs. YOLOv8-l: 43.7 M parameters, 165.7 GFLOPs), meaning that training multiple YOLOv11 configurations required substantially less time and memory than their YOLOv8 equivalents. However, as demonstrated, this computational efficiency during training did not translate to improved detection accuracy for this microcarrier detection task.

**TABLE 6:** Computational efficiency comparison of evaluated architectures (in bold the best performing model based on mAP_50_)**.**

Model	Parameters (M)	GFLOPs	Latency (ms)	FPS	GPU Memory (GB)
YOLOv8-n	3.16	8.9	20.0 ± 0.5	50.0	0.07
YOLOv8-s	11.17	28.8	38.0 ± 0.7	26.3	0.11
YOLOv8-m	25.90	79.3	80.1 ± 0.6	12.5	0.19
YOLOv8-l	**43.69**	**165.7**	**137.1 ± 0.9**	**7.3**	**0.28**
YOLOv8-x	68.23	258.5	207.8 ± 1.1	4.8	0.40
YOLOv11-n	2.62	6.6	19.4 ± 0.5	51.6	0.10
YOLOv11-s	9.46	21.7	35.4 ± 0.7	28.3	0.14
YOLOv11-m	20.11	68.5	80.3 ± 0.7	12.5	0.21
YOLOv11-l	25.37	87.6	100.7 ± 0.8	9.9	0.24
YOLOv11-x	56.97	196.0	197.5 ± 1.1	5.1	0.41
RT-DETR-l	32.97	108.3	170.3 ± 1.1	5.9	0.28
RT-DETR-x	67.47	232.7	300.1 ± 1.4	3.3	0.44

The model shown in bold denotes the one selected as best-performing according to the SHA methodology applied in the model evaluation.

Regarding inference, all evaluated models process a single image in well under one second, which is entirely sufficient for the intended laboratory monitoring application where bioreactor samples are acquired at intervals of several minutes. The focus of computational efficiency reporting in this study is therefore primarily on training feasibility rather than real-time inference requirements. Compared to manual quantification time of approximately 10 min per image, any of the evaluated models represents a dramatic reduction in analysis time, effectively eliminating a repetitive bottleneck from the operator’s daily workflow regardless of the specific inference latency.

Notably, YOLOv11-l offered a favorable efficiency profile, achieving 9.9 FPS with 42% fewer parameters (25.4 M vs. 43.7 M) and 47% fewer GFLOPs (87.6 vs. 165.7) than YOLOv8-l. For applications where computational resources are more constrained than in our setup, or where slightly lower accuracy is acceptable, YOLOv11-l could represent a reasonable alternative. This highlights that model selection involves trade-offs between accuracy, speed, and resource consumption that must be evaluated in the context of specific deployment requirements.

RT-DETR variants exhibited the highest latencies among comparable model sizes (RT-DETR-l: 170 ms vs. YOLOv8-l: 137 ms), compounding their accuracy disadvantage with inferior computational efficiency. The transformer-based architecture’s attention mechanisms, while powerful for large-scale datasets, introduce computational overhead that is not justified by improved performance in this small-dataset regime.

**FIGURE 10:**
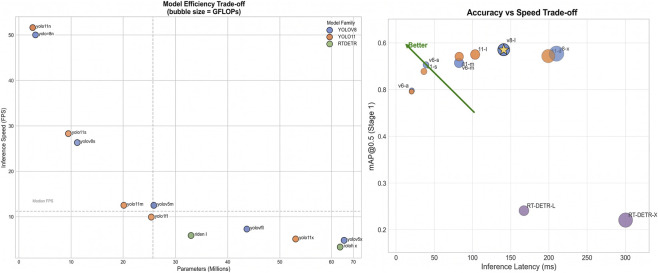
Computational efficiency trade-off analysis of evaluated architectures. (Left) Inference speed (FPS) versus model size (parameters in millions) for all 12 candidate models. Bubble size represents computational complexity (GFLOPs). Dashed lines indicate median values for reference. YOLOv11 variants achieve higher FPS than YOLOv8 counterparts at equivalent parameter counts, reflecting architectural efficiency improvements. RT-DETR models cluster in the lower-right region, indicating high parameter counts with comparatively low throughput. (Right) Detection accuracy (mAP_50_ at Stage 1) versus inference latency. The arrow indicates the direction of optimal performance (high accuracy, low latency). YOLOv8-l (★) achieves the best accuracy among all models at moderate computational cost, while RT-DETR variants exhibit both the lowest accuracy and highest latency, making them unsuitable for this application. All measurements performed on NVIDIA T400 (4 GB VRAM) at 640 × 640 input resolution with FP16 precision.

GPU memory consumption remained modest across all architectures (<0.5 GB), well within the 4 GB capacity of the T400 GPU. This low memory footprint suggests that deployment on even more resource-constrained hardware (e.g., embedded systems, older GPUs) is feasible, potentially enabling integration with existing laboratory equipment without dedicated computational infrastructure (Figure 10).

## 4 Discusion

The results of this study provide insights that are valuable for the broader implementation of deep learning–based object detection in bioprocess monitoring. These insights go beyond the specific task of microcarrier colonization assessment and inform general considerations for integrating image based analytics into bioprocess workflows.

### 4.1 Newer model versions do not necessarily guarantee domain-specific improvements

A central finding of this work is that YOLOv8-l consistently and substantially outperformed YOLOv11-l across all evaluation stages, with the performance gap widening under extended training (final mAP_50_–_95_: 0.856 vs. 0.790). This result contradicts the expectation, based on general-domain benchmarks such as COCO, that architectural updates in YOLOv11 would translate to improved performance across applications (Khanam and Hussain, 2024).

Several factors may explain this discrepancy. First, the architectural refinements in YOLOv11, including C3k2 blocks and C2PSA attention modules, were optimized for diverse natural image datasets characterized by high inter-class variability, complex backgrounds, and objects spanning multiple scales. In contrast, microcarrier detection presents a comparatively homogeneous task: objects exhibit uniform circular morphology, consistent size distribution, and appear against relatively uniform backgrounds. Under these conditions, the additional representational capacity of YOLOv11 may offer limited benefit while potentially introducing optimization challenges.

Second, attention mechanisms such as C2PSA typically require substantial training data to learn effective feature weighting patterns (Li et al., 2020). With fewer than 699 images, our dataset may be insufficient for YOLOv11 to fully exploit its architectural innovations. This interpretation is consistent with the observation that the performance gap between YOLOv8 and YOLOv11 narrowed at intermediate training budgets (Stage 2) before widening again under extended training, a pattern that could reflect suboptimal attention learning dynamics in YOLOv11.

These findings underscore the importance of empirical validation when adopting new model architectures for domain-specific applications. Under the evaluated conditions, the assumption that ‘newer is better’ was not supported in this specialized biomedical context, though further validation on larger and more diverse datasets would be needed to draw general conclusions.

### 4.2 Transformer-based detection may require larger datasets in domain-specific contexts

The poor performance of RT-DETR variants (mAP_50_ < 0.25) provides a cautionary note regarding the application of transformer-based detectors to small-scale biomedical datasets. Despite achieving competitive results on COCO with over 100,000 training images, RT-DETR failed to learn effective representations from our 699-image dataset (Ma et al., 2022).

This outcome is consistent with the broader understanding that transformers are data-hungry architectures that typically underperform CNNs in low-data regimes (Ma et al., 2022). The self-attention mechanism in RT-DETR’s hybrid encoder must learn to weight spatial relationships across feature maps, a task that requires exposure to diverse training examples to avoid overfitting spurious patterns. In contrast, CNN-based architectures like YOLO benefit from strong inductive biases (locality, translation equivariance) that facilitate learning from limited data, particularly when initialized from pretrained weights.

For practitioners in biomedical imaging, where annotated datasets are often small and expensive to acquire, these results suggest that CNN-based detectors may represent a more pragmatic starting point. The poor performance of RT-DETR in our setting is consistent with the known data requirements of transformer-based architectures reported in the literature, though we acknowledge that this conclusion is drawn from a specific imaging context with a relatively small dataset. As the field moves towards larger annotated collections and domain-specific pretraining strategies, transformer-based detectors may well become competitive alternatives for biomedical microscopy tasks—a question that remains open and worth exploring in future work.

### 4.3 Threshold calibration is essential for application alignment

An important methodological contribution of this work is the demonstration that raw detection metrics (mAP) do not fully capture downstream task performance. The threshold calibration procedure, which optimized confidence and IoU thresholds to minimize colonization estimation error rather than maximize detection metrics, yielded an operating point (confidence = 0.55, IoU = 0.4) substantially different from default values.

This calibration reduced the validation MAE from the raw detection baseline to 10.71%, highlighting the importance of application-specific tuning. The resulting precision-recall trade-off (82.3% precision, 76.2% recall on test set) reflects a deliberate choice to accept some missed detections in exchange for more accurate colonization percentage estimation. This trade-off would be suboptimal for applications requiring exhaustive object enumeration but is well-suited for percentage-based monitoring where proportional under-detection of both classes has limited impact on the final ratio.

Practitioners deploying detection models for quantitative bioprocess monitoring should consider similar calibration procedures, recognizing that the optimal operating point depends on the specific application requirements rather than generic detection benchmarks.

### 4.4 Practical implications for laboratory deployment

From a practical standpoint, the performance achieved, MAE of 11.75% with processing time of seconds per image, represents a substantial improvement over manual quantification. Laboratory technicians typically require approximately 10 min to evaluate a single image, and inter-operator variability can exceed 15% depending on training and fatigue counting (Cai et al., 2024; Salinas et al., 1997). The automated pipeline thus offers: (i) approximately 100-fold reduction in analysis time, (ii) complete elimination of operator fatigue effects, (iii) objective and reproducible quantification, and (iv) potential for real-time monitoring integration.

The systematic underestimation bias of −5.28% represents a known, consistent offset that could be corrected through post hoc calibration if absolute accuracy is required. Alternatively, for trend monitoring applications where relative changes matter more than absolute values, the bias is largely inconsequential as long as it remains stable across measurements.

Although current computational standards often exceed the specifications of an NVIDIA T400 (4 GB VRAM), the results obtained and the methodology proposed in this work indicate that it is possible to achieve solid performance using conventional laboratory computers equipped with mid-range GPUs, without the need for specialized infrastructure, which reinforces the practical applicability and ease of deployment of the proposed approach. It is worth noting that computational efficiency in this context is primarily relevant for the training and model comparison process rather than for inference during routine laboratory use. All evaluated models process a single image in well under one second, which is entirely sufficient given that bioreactor samples are typically acquired at intervals of several minutes. The key practical advantage of using computationally efficient architectures lies in making the multi-architecture comparative evaluation feasible on standard laboratory hardware, reducing training times and enabling the exploration of multiple configurations without requiring dedicated high-performance computing infrastructure.

### 4.5 Limitations

Several limitations should be acknowledged when interpreting these results.

First, the dataset originated from a single laboratory using specific microscopy equipment (fluorescence-inverted microscope), dyes and staining protocols (fluorescein diacetate), and microcarrier types. Generalization to other facilities, equipment configurations, or cell types would require validation studies and potentially domain adaptation or fine-tuning. The extent to which the trained model transfers across laboratories remains an open empirical question.

Second, the sample size of 699 images, while sufficient for CNN-based detection, limited our ability to evaluate transformer architectures and may have constrained the performance ceiling of all models. Larger datasets might reveal different relative rankings among architectures or enable more sophisticated approaches such as ensemble methods.

Third, ground-truth annotations were generated through expert manual labeling, which itself carries inherent subjectivity. Although five experts participated in annotation and consensus protocols were employed, some degree of label noise is inevitable. This noise establishes an effective upper bound on achievable model performance that cannot be exceeded regardless of architectural improvements.

Fourth, the proprietary nature of this research prevents the public release of fluorescence microscopy images, trained model weights, and annotations. While an anonymized version of the analysis code has been made available and full methodological detail is reported in this manuscript, the absence of publicly available data limits the ability of the broader research community to directly validate or build upon these findings.

Fifth, the SHA-based model selection strategy, while efficient and principled, carries inherent limitations. The use of uniform hyperparameters across all candidate architectures means that individual models may not have been evaluated at their optimal configuration, and the fixed training budget may place certain architectures at a disadvantage, particularly those that require more epochs to converge such as transformer-based models. While the consistency of rankings across SHA stages provides indirect validation of the approach, independent hyperparameter tuning of each architecture could potentially alter the relative performance rankings. Future work could explore more exhaustive optimization strategies, ideally on larger computational infrastructure, to complement the findings reported here.

Sixth, while the use of a modest GPU represents a limitation in terms of maximum achievable speed, the model nevertheless demonstrated robust computational efficiency under these constrained conditions—an encouraging result for practical implementation. Although higher-end hardware would provide faster inference at increased cost, the trade-off between hardware investment and performance gains was not systematically evaluated.

Finally, the current pipeline provides static, per-image colonization estimates. Integration with time-series analysis for colonization kinetics modeling, or with bioreactor control systems for closed-loop optimization, represents natural extensions that were beyond the scope of this work.

### 4.6 Considerations for production deployment

While the achieved MAE supports research and development applications, deployment in regulated manufacturing environments, particularly those operating under Good Manufacturing Practice (GMP) guidelines requires additional considerations. Current regulatory frameworks from agencies such as the FDA and EMA emphasize the need for validated, traceable, and auditable analytical methods in critical process steps (FDA, 2015; ICH, 2026). Automated image analysis systems, despite their advantages in speed and reproducibility, introduce challenges related to algorithmic transparency, validation protocols, and accountability for analytical decisions.

We therefore recommend that production deployment adopt a Human-In-The-Loop (HITL) paradigm, where the deep learning system serves as a decision-support tool rather than a fully autonomous replacement for human operators. In this configuration, the algorithm provides rapid preliminary quantification and flags images with high uncertainty or unusual characteristics, while trained personnel retain responsibility for final verification and release decisions. This hybrid approach preserves the efficiency benefits of automation while maintaining the human oversight required for GMP compliance and regulatory defensibility.

Such integration also facilitates continuous model improvement through operator feedback, enabling detection of distribution shifts (i.e., changes in staining protocols, new microcarrier batches) that might degrade model performance over time. The systematic collection of operator corrections creates a natural mechanism for ongoing validation and potential model retraining when performance degradation is detected.

## 5 Conclusion

This study presented a comparative evaluation of three state-of-the-art object detection architectures, YOLOv8, YOLOv11, and RT-DETR, for automated quantification of mesenchymal stromal cell colonization on microcarriers in bioreactor cultures. Using a dataset of 699 fluorescence microscopy images containing 46,982 annotated microcarriers, we evaluated 12 model configurations through a Successive Halving Algorithm (SHA) that progressively allocated computational resources to the most promising candidates.

The main findings can be summarized as follows:

- YOLOv8-l emerged as the best-performing architecture, achieving mAP_50_ of 0.866 on validation and 0.796 on the test set, with a colonization estimation MAE of 11.75%. This performance substantially exceeds that of YOLOv11-l (mAP_50_ = 0.797) despite YOLOv11 incorporating more recent architectural innovations.
- Newer model versions do not guarantee improved domain-specific performance in our conditions. The consistent superiority of YOLOv8 over YOLOv11 across all model sizes and training budgets demonstrates that architectural improvements optimized for general-domain benchmarks do not automatically transfer to specialized biomedical applications.
- Transformer-based detectors (RT-DETR) underperformed substantially (mAP_50_ < 0.25), highlighting the data-hungry nature of attention-based architectures and reinforcing the pragmatic advantage of CNN-based approaches for small-scale biomedical datasets.
- Application-specific threshold calibration is essential to align detection performance with downstream task requirements. The optimized operating point (confidence = 0.55, IoU = 0.4) differed substantially from default values and was critical for minimizing colonization estimation error.

From a practical standpoint, the developed pipeline reduces image analysis time from approximately 10 min (manual) to seconds (automated), representing a ∼100-fold improvement in throughput while eliminating operator-dependent variability. The modest computational requirements (NVIDIA T400, 4 GB VRAM) support deployment of standard laboratory infrastructure without specialized hardware.

In conclusion, this work demonstrates that automated deep learning-based quantification of microcarrier colonization is technically viable and practically beneficial, while highlighting the importance of empirical architecture validation and the need for thoughtful integration strategies in regulated manufacturing contexts.

## Data Availability

The datasets generated and/or analyzed during the current study are not publicly available due to proprietary restrictions, intellectual property considerations, and confidentiality obligations. The fluorescence microscopy images, associated annotations, source code, and trained model weights are subject to the same restrictions and therefore are not available in public repositories. An anonymized version of the analysis code is publicly available at “Automated-Microcarrier- Bioreactor-detection/docs/METHODOLOGY.md at main ⋅ manutfds/Automated-Microcarrier- Bioreactor-detection ⋅ GitHub” Requests for additional information regarding the methodology or potential academic collaboration should be directed to the corresponding authors.
